# Toxic encephalopathy due to self-administration of over-the-counter amantadine: a case report of a patient undergoing maintenance hemodialysis and safety alert

**DOI:** 10.3389/fmed.2026.1825103

**Published:** 2026-05-11

**Authors:** Junqi Hu, Yuanzi Liang, Kebing Sun, Yuefei Xiao

**Affiliations:** 1 Department of Nephrology, Aerospace Center Hospital, Beijing, China; 2 Department of Radiology, Aerospace Center Hospital, Beijing, China

**Keywords:** amantadine, chronic kidney disease, hemodialysis, hemoperfusion, public health

## Abstract

This report describes a 49-year-old male patient with end-stage renal disease who developed acute mania, incoherent speech, and vivid visual hallucinations after self-administering an over-the-counter cold medication containing amantadine during regular hemodialysis. Laboratory toxicology analysis confirmed toxic encephalopathy caused by significantly elevated blood amantadine levels. Following five consecutive days of combined hemoperfusion and hemodialysis for intensive clearance, the patient achieved complete resolution of neuropsychiatric symptoms. This case highlights the unique high-risk profile of chronic kidney disease (CKD) patients, particularly dialysis-dependent individuals, when engaging in self-medication. It underscores the critical importance for clinicians to proactively inquire about medication history, understand relevant pharmacokinetic alterations, and implement targeted interventions. While single-case evidence cannot drive regulatory change, this case suggests a potential safety gap in regions where amantadine remains available over-the-counter, warranting further pharmacoepidemiologic investigation.

## Case report

Patient is a 49 year old male who undergoes outpatient hemodialysis regularly for 5 years due to chronic kidney disease stage 5. Past medical history includes hypertension, type 2 diabetes, and chronic heart failure.Long-term oral medications included only roxadustat 100 mg capsules taken three times a week and lanthanum carbonate 500 mg chewable tablets taken three times a day. After initiation of regular maintenance hemodialysis, his blood pressure, volume status, and blood glucose levels became adequately controlled without the need for additional pharmacotherapy for hypertension, diabetes, or heart failure. Patient was admitted for “sudden agitation and incoherent speech for 3 days.” Family members reported that three days prior, without apparent cause, the patient exhibited abnormal mental and behavioral changes, presenting as emotionally agitated and incoherent speech. He claimed to see “many small people in the air constantly throwing apples at him.” Approximately one week before symptom onset, the patient self-diagnosed a “cold” due to nasal discharge and fatigue. He purchased “Compound Amantadine Hydrochloride Tablets” (containing amantadine 100 mg, paracetamol 250 mg, caffeine 15 mg, chlorpheniramine maleate 2 mg, and artificial bezoar 10 mg per tablet) from a local pharmacy. He took one tablet one time daily for 10 consecutive days without consulting a physician or pharmacist.

Admission examination: Blood pressure 146/66 mmHg, heart rate 96 beats/min. Patient was delirious and agitated. Specialized neurological examination revealed no significant positive findings. Table 1 provides detailed laboratory results. No significant abnormalities were observed on the head CT scan.

**Table 1:** Relevant assistant examinations upon admission.

	Parameter	Result
**Biochemistry**	Cr	1,016.5 μmol/L
	BUN	16 mmol/L
	eGFR	4.59 mL/min/1.73 m^2^
	Glu	5.2 mmol/L
	K	3.72 mmol/L
	Ca	2.46 mmol/L
	P	1.17 mmol/L
	Alb	40 g/L
	ALT	12.3 U/L
	AST	29.7 U/L
	TB	12.1 μmol/L
	DB	2.2 μmol/L
	IDB	9.9 μmol/L
	LDH	329.6 U/L
**Complete blood count**	WBC	7.74 × 10^9^/L
	NE%	84.7%
	Hb	108 g/L
	PLT	197 × 10^9^/L
	CRP	6.14 mg/L
**Other**	CK-MB	5.4 ng/mL
	APTT	31.7 s
	DD	342 μg/L
	INR	1

Given the highly suggestive history of drug-related encephalopathy, blood toxicology testing was initiated. Plasma amantadine concentration was measured by high-performance liquid chromatography-tandem mass spectrometry (HPLC-MS/MS) at the authorized third-party clinical toxicology testing laboratory in Beijing. The result was 3.2 μg/mL (toxic >2.0 μg/mL).This confirmed toxic encephalopathy due to amantadine accumulation.

Upon diagnosis, the suspected medication was immediately discontinued. To rapidly eliminate accumulated amantadine, an intensive treatment regimen combining hemoperfusion with serial hemodialysis was initiated. This protocol was performed daily for 5 consecutive days. Within 24 h of treatment initiation, the patient's agitation showed marked improvement. By the third day of therapy, visual hallucinations resolved and consciousness gradually cleared. After completing the five-day course, the patient's mental status and behavior fully normalized, cognitive function returned to baseline levels, and the patient was discharged successfully. Comprehensive medication safety education was provided to the patient and family upon discharge. Timeline of clinical events presented in Table 2.

**Table 2:** Timeline of key clinical events.

Time point	Clinical event
10 days before admission	Self-administered OTC amantadine for cold symptoms
3 days before admission	Onset of agitation, incoherent speech, and visual hallucinations
Admission	Diagnosed with amantadine-induced toxic encephalopathy; started hemoperfusion + hemodialysis
Day 1 of treatment	Agitation markedly improved
Day 3 of treatment	Visual hallucinations resolved; consciousness cleared
Day 5 of treatment	Neuropsychiatric symptoms fully normalized; patient discharged

## Discussion

For any CKD patient presenting with acute neuropsychiatric symptoms, a thorough and targeted medication history inquiry is the first and critical step in diagnosis. This inquiry must extend beyond prescription drugs to proactively and specifically cover all over-the-counter medications, Chinese herbal medicines, health supplements, and “folk remedies.” Many patients are unaware that these products are also “medications” or that their impaired renal function fundamentally alters the safety profile of these substances. The diagnosis in this case directly relied on the crucial clue provided by the family: “taking cold medicine.” Amantadine is almost entirely cleared by the kidneys in its unchanged form. This means that in patients with impaired renal function, daily dosing at standard levels leads to linear accumulation of the drug in the body, rapidly reaching toxic concentrations (1–4). Therefore, clinicians must assess the estimated glomerular filtration rate (eGFR) before initiating any new medication in CKD patients and strictly adhere to dosage adjustment guidelines for renal impairment. Many common drugs are contraindicated in severe renal insufficiency. For amantadine poisoning with severe neuropsychiatric symptoms, actively accelerating drug clearance is crucial alongside supportive care. Amantadine exhibits a protein binding rate of 67% (5). Previous studies indicate that hemodialysis has negligible clearance efficacy for amantadine, with a 4-h hemodialysis session removing less than 5% of the drug (1). Hemoperfusion followed by hemodialysis achieves more efficient and sustained toxin clearance through synergistic adsorption and diffusion effects. This combination is currently the recommended effective blood purification modality for treating such poisoning. Treatment typically requires consecutive days until significant clinical improvement is observed.

The causal relationship between amantadine self-administration and the development of acute toxic encephalopathy in this patient was evaluated using the WHO-UMC causality assessment system. The assessment yielded a rating of Probable/Likely, based on the following: (A): a reasonable temporal sequence – neuropsychiatric symptoms emerged after initiation of amantadine-containing cold medication, which is pharmacologically plausible given the drug's marked accumulation in end-stage renal disease; (B): exclusion of alternative causes – uremic, infectious, cerebrovascular, and hepatic encephalopathies were systematically ruled out by normal laboratory and imaging findings, and concomitant medications (roxadustat, lanthanum carbonate) were not associated with such symptoms; (C): positive dechallenge – after discontinuation of amantadine and initiation of daily combined hemoperfusion and hemodialysis, agitation improved within 24 h, visual hallucinations resolved by day 3, and full neurologic recovery occurred by day 5. Rechallenge was not performed for ethical reasons. Therefore, according to WHO-UMC criteria, amantadine accumulation is the probable cause of this patient's toxic encephalopathy.

This case, while limited to a single patient, highlights a potential safety concern in regions where amantadine remains available over-the-counter (e.g., China). Millions of CKD patients worldwide exist, and many are unaware of their declining kidney function. They may be susceptible to accessing OTC medications with potential neurotoxicity. However, we acknowledge that case reports cannot establish population-level risk. Future pharmacoepidemiologic studies should quantify the incidence of amantadine toxicity in CKD patients using OTC products. Pending such evidence, we suggest that clinicians in these regions maintain a low threshold for inquiring about OTC cold medicine use in dialysis-dependent patients and consider routine medication safety education as part of standard CKD care. Regulatory authorities may wish to reassess the OTC status of drugs like amantadine that rely heavily on renal excretion and have narrow therapeutic windows, or add prominent warnings on packaging regarding renal impairment.

Several limitations warrant consideration. First, this is a single case without rechallenge, so causality cannot be proven definitively. Second, medication history relied on family report, introducing potential recall bias. Third, the generalizability of our public health observations is limited by regional differences in OTC regulations. Nevertheless, the strong temporal relationship, toxic plasma level, and clinical response to enhanced clearance support the causal role of amantadine.

In summary, amantadine can cause life-threatening toxic encephalopathy in patients with severe renal impairment. Successful management requires vigilance, prompt toxicological diagnosis, and aggressive blood purification. Targeted interventions are needed to protect vulnerable CKD populations from preventable OTC drug-related harm in regions where amantadine is available without a prescription.

## Data Availability

The original contributions presented in the study are included in the article/supplementary material, further inquiries can be directed to the corresponding author.
